# Liujunanwei decoction attenuates cisplatin-induced nausea and vomiting in a Rat-Pica model partially mediated by modulating the gut micsrobiome

**DOI:** 10.3389/fcimb.2022.876781

**Published:** 2022-08-19

**Authors:** Dongmei Chen, Yi Guo, Yufei Yang

**Affiliations:** ^1^ Department of Integrative Oncology, China-Japan Friendship Hospital, Beijing, China; ^2^ Department of Gastroenterology, Dongzhimen Hospital, Beijing University of Chinese Medicine, Beijing, China; ^3^ Department of Oncology, Xi-Yuan Hospital, China Academy of Chinese Medical Sciences, Beijing, China

**Keywords:** liujunanwei decoction, traditional chinese medicine, chemotherapy induced nausea and vomiting, intestine, microbiome, serotonin

## Abstract

Studies show that traditional Chinese medicine (TCM), such as Liujunanwei (LJAW) decoction, can play important roles in alleviating side effects of chemotherapy. The purpose of this study was to understand how LJAW can counter chemotherapy-induced emesis *via* alteration of gut microbiota. We evaluated the effect of LJAW on cisplatin (DDP)-induced nausea and vomiting using a rat-pica model. Rats react to emetic-producing stimuli with increased kaolin consumption, a phenomenon called pica. The rats were injected with cisplatin and then randomly assigned to the control (DDP), Ondansetron or LJAW. The intake of kaolin and chow diet as well as body weights were recorded every 24 hours. Fecal samples were collected prior to, after three and seven days of treatment. The expression of proteins was measured by western blot. The concentration of cytokines and serotonin was evaluated using ELISA assay kits. Kaolin consumption in rats induced by cisplatin was reduced by 16.5%, 22.5%, and 30.1% in the LJAW group compared to the DDP group at 24 hours, 48 hours and 72 hours, respectively (*p*>0.05). LJAW significantly increased the food intake of the rats (13.94 ± 4.73 g) during the first 24 hours as opposed to the DDP (9.23 ± 3.77 g) (*p*<0.05). 16S rRNA gene sequencing showed the abundance of *Bacteroidetes* increased in cisplatin treated rats. In addition, cisplatin injection caused an enrichment of *Escherichia-Shigella and Enterococcu*s at the genus level. While, enrichment of *Blautia* and *Lactobacillus* was presented in LJAW treated rats. Serotonin decreased in LJAW treated intestine and medulla oblongata tissues. Further, the protein expression of tryptophan hydroxylase 1 (TPH1) a rate limiting enzyme of serotonin was inhibited in LJAW treated rat’s jejunum compared with cisplatin only treated rats. In addition, LJAW downregulated chemotherapy induced elevated inflammation. The results of this study indicated that LJAW is capable of decreasing cisplatin-induced kaolin intake in rat-nausea model (pica), which might be mediated through gut microbiome-induced anti-inflammation and anti-serotonin synthesis functions.

## Introduction

Chemotherapy is one of the most widely used methods in cancer treatment. Chemotherapy-induced nausea and vomiting (CINV) is the most common side effect in patients undergoing chemotherapy, the incidence of CINV is as high as 65%-85% (Patel et al., 2019), especially those undergoing highly emetogenic regimens such as anthracycline combined with cyclophosphamide. Although the administration of neurokinin-1 receptor antagonists or type three-hydroxytryptamine (5-HT3) antagonists already decreases the incidence of vomiting, CINV still remains an issue for many patients (Gupta et al., 2021). At the same time, anti-nausea and vomiting agents might cause constipation as they would inhibit the movement of gastrointestines. Additionally, there are various unmet needs while patients receiving chemotherapy, such as the management of non-acute forms of CINV, the identification and management of patients prone to CINV and gastrointestinal symptoms including loss of appetite. Traditional Chinese medicine (TCM), such as ginger and *Forsythia viridissima*, has been reported to be effective in treating CINV (Ryan et al., 2012; Yi et al., 2019). Liujunanwei granule (LJAW) is a TCM formula that has been used along with chemotherapy to alleviate CINV. Our retrospective study revealed that the response of CINV is 87.5% in colorectal cancer patients treated with LJAW when undergoing the Xelox or FOLFOX regimens (Yan et al., 2020). However, the underlying mechanism remains unknown.

Both peripheral and central nervous system (CNS) pathways with different mechanisms are involved in acute CINV and delayed CINV (Janelsins et al., 2013; Singh et al., 2016). It’s been reported that free radicals generated by chemotherapeutic agents would damage the barrier of intestine and cause the release of serotonin by enterochromaffin cells in jejunum (Janelsins et al., 2013; Rapoport, 2017). Serotonin binds to intestinal vagal afferent nerves *via* 5-HT3 receptors, which trigger the vomiting reflex *via* the nucleus of the solitary tract and chemoreceptor trigger zone in the CNS (Rapoport, 2017). For delayed CINV, substance P has been regarded as the principal neurotransmitter. In recent years, as the development of the next generating sequencing, numerous studies have shown that gut microbiome participated in CINV. Chemotherapeutic agents would influence the gut-brain axis *via* altering gut microbiome composition as well as functions (Song and Bai, 2021). Dysbiosis of intestine microbiome impairs the gut lining and further stimulate enterochromaffin cells inducing the release of serotonin (Bajic et al., 2018). Meanwhile, dysbiosis of intestine microbiome can also activate inflammatory cells such as macrophages and T lymphocytes, which induce the pro-inflammatory cytokines or chemokines (Jordan et al., 2018; Zhong et al., 2019). Those studies indicated that gut microbiome could directly or indirectly contribute to CINV.

TCM is featured by oral administration, it will interact with gut microbiome inevitably. Previous investigations have shown that TCM is able to maintain the homeostasis of intestine microbiome (Chang et al., 2015; Zhou et al., 2016), and the gut microbiome could also exert pharmacological effects of TCM on host (Park et al., 2006). Here, we investigated the possible mechanisms involved in the anti-CINV efficacy of LJAW *via* the intestinal microbiome.

## Materials and methods

### Preparation of LJAW

The component herbs of LJAW used in this study are as follows: *Pseudostellariae Radix, Poria, Macrocephalae Rhizoma, Radix Rhizoma Glycyrrhizae, Pinelliae rhizome, Citrus reticulata Blanco, Galli Gigerii Endothelium Corneum, Massa Medicata Fermentata, Setariae Fructus Germinatus, Hordei Fructus Germinatus, Crataegi Fructus, Amomi Fructus*, and *Aucklandiae Radix* at a weight ratio of 15:5:5:3:5:5:5:5:5:5:3:3. The LJAW used in the study were purchased from Beijing Tcmages Pharmaceutical Co., Ltd. (Beijing, China). Prior to each experiment, the formula LJAW were milled to powders and dissolved in filtered deionized water. The concentrations of LJAW in the study refer to the crude drug concentrations.

### Laboratory animal studies

Eight-week-old female Wistar rats weighing 180 ± 20 g was used. The animal facility was kept at 23°C and 10% humidity, with a cycle of 12 hours of light and 12 hours of dark. The rats were acclimated for 1 week in the facility prior to the experiment.

Pica (eating nonnutritive substances such as kaolin) in rats, analogous to vomiting in animals that developed emetic reflex, is regarded as an alternative model for nausea and vomiting in rodents (Sharma et al., 1997). To determine the efficacy of LJAW in treating CINV, rats were injected with cisplatin intraperitoneally at a dose of 6 mg.kg^-1^ (cisplatin-induced pica) and then randomly assigned to DDP, LJAW and ondansetron. The rats were gavage with distilled water (10 ml.kg^-1^), LJAW (12.78 g.kg^-1^) or ondansetron (2.6 mg.kg^-1^) for 7 days. Another group of rats were set as vehicle-control without any treatment. The dose of LJAW is equal to that used in humans in the clinic. The dose of LJAW was choose based on the results of our preliminary experiment. Briefly, we treated rats with high-, medium- and low-dose of LJAW. Clinically relevant dose was set as medium-dose, 2-times clinically relevant dose was set as high-dose, and 0.5-time of clinically relevant dose was set as low-dose. Preliminary data showed that medium dose of LJAW exerted the best therapeutic effect. Kaolin and chow intake as well as body weight were monitored every day. Feces were collected prior to cisplatin administration, 3 and 7 days after cisplatin injection. The rats were euthanized, and the intestine and medulla oblongata were resected 3 and 7 days after cisplatin injection, respectively. The samples of jejunum, medulla oblongata and feces were flash frozen in liquid nitrogen and stored at -80°C. Part of the jejunum tissues was fixed in a 10% formalin-PBS solution for hematoxylin-eosin staining (HE) and immunohistochemistry staining (IHC).

### Western blotting

Both intestine and medulla oblongata tissues were lysed in ice-cold lysis buffer (Thermo Fisher Scientific, Waltham, MA) and homogenized with a tissue homogenizer (FLUKO Shanghai Equipment, Shanghai) followed by centrifugation at 10,000 g for 10 minutes at 4°C. Protein levels were quantified using a bicinchoninic acid (BCA) protein assay kit (cwbiotech, Beijing). An equal amount of protein (20 µg) was applied to a 10% to 15% SDS gel and then transferred onto polyvinyl membranes according to standard procedure. We blocked the membranes with 5% nonfat dry milk blocking buffer prepared in Tris-buffered saline with 0.1% Tween 20 for 1 hour at room temperature. The membranes were then probed with primary antibodies against NF-κB p65 (Cell Signaling Technology, Danvers, MA), mdy88 (Cell Signaling Technology, Danvers, MA), TLR4(Cell Signaling Technology, Danvers, MA), TPH1(Cell Signaling Technology, Danvers, MA) or beta-actin (Cell Signaling Technology, Danvers, MA) overnight at 4°C. The membranes were washed and incubated with secondary antibodies (anti-rabbit IgG) prepared in 5% nonfat dry milk blocking buffer with 0.1% Tween 20 for 1 hour at room temperature. The membranes were then washed again and incubated with the ECL+ detection kit for 5 minutes. Then, lights were turned off, and the protected membrane was exposed to the X-ray film. The exposure time varied according to the bands. Gel Imagestem ver. 4.00 (Tanon, Beijing) was used for protein band quantification.

### ELISA Assay Kit

5-HT and cytokine levels in jejunum and medulla oblongata tissues were measured by Elisa assay kit. About 30mg of tissue for each sample was taken from the above-mentioned sites after their dissection. Samples were rinsed with ice-cold PBS and dried with filter paper to remove feces or blood and weighted. Tissues was homogenized in PBS(1:10) by tissue homogenizer and followed by sonicating by using 2 cycles of 30s each to break cell membranes. Then, homogenates were centrifugated for 15 min at 5000rpm and the supernatants were collected and used for Elisa analysis.

5-HT(Cambridge, MA) and cytokines levels including IL-6, IL-1β, IL-10, TNF-α and TGF-β(Elabscience, Wuhan) were measured by ELISA according to the manufacturer’s instructions. Then, the concentration of the samples was calculated according to the OD value.

### Immunohistochemistrystaining

Formalin-fixed, paraffin-processed jejunum tissues were used for biomarker identification using IHC staining. The slides were baked at 60°C for over 2 hours and then deparaffinized and rehydrated. The antigens were unmasked by heat-induced antigen retrieval. The slides were then immersed in 3% H_2_O_2_-methanol solution followed by blocking with 5% goat serum in 0.3% Triton X-100 PBS. The slides were stained with ZO-1(proteintech, Wuhan) and occludin(proteintech, Wuhan) antibodies in a humidified chamber overnight at 4°C, washed three times with PBS and further incubated with secondary antibody at room temperature for 45 minutes. The slides were then incubated with avidin-biotin complex (Vector Laboratories, Burlingame, CA) followed by DAB substrate for antibody visualization and counterstained with Mayer’s hematoxylin, dehydrated, and mounted with ClearMount mounting medium (American MasterTech, Lodi, CA) (Pan et al., 2015).

### DNA extraction and bacterial 16Sv4 rRNA gene sequencing

16S rRNA sequencing was carried out by Beijing Guoke Biotechnology. Total genome DNA from samples was extracted using CTAB/SDS method.16S rRNA genes were amplified used the specific primer with the barcode. Then, mix same volume of 1X loading buffer (contained SYB green) with PCR products and operate electrophoresis on 2% agarose gel for detection. Samples with bright main strip between 400-450bp were chosen for further experiments. Then, mixture PCR products was purified with GeneJET Gel Extraction Kit (Thermo Scientific). Sequencing libraries were generated using Illumina TruSeq DNA PCR-Free Library Preparation Kit (Illumina, USA) following manufacturer’s recommendations and index codes were added. The library quality was assessed on the Qubit@ 2.0 Fluorometer (Thermo Scientific) and Agilent Bioanalyzer 2100 system. At last, the library was sequenced on an Illumina HiSeq platform and 250 bp paired-end reads were generated.

Raw sequence data were analyzed using the QIIME 2 pipeline (https://qiime2.org/) (version 2020.2). Raw sequence data were denoised using DADA2 in the QIIME 2 package. The paired-end FASTQ files were processed by Phred quality score-based quality filtering, merging of the paired ends, chimera removal, singleton removal, and construction of a feature table consisting of amplicon sequence variants (ASV). Using DADA2 denoise-single method, we removed low quality regions of the sequences. All ASV were aligned to mafft through q2 alignment, and phylogenetic analysis was performed using fasttree2. A scikit-learn naïve Bayes machine-learning taxonomy classifier against the SILVA (https://www.arb-silva.de/) 16S rRNA Version 138 reference sequences was trained with the q2-feature-classifier plugin using the V3-V4 regions of 16S rRNA sequences. Taxonomy was assigned to each ASV using the ‘classifysklearn’ command in q2-feature-classifier. The α-diversity was calculated between groups using the Kruskal-Wallis pairwise test and permutational multivariate analysis of variance, respectively. In order to compute α-diversity, we rarify the ASV table and calculate Chao1 to estimates the species abundance. We used unweighted unifrac for Principal Coordinate Analysis (PCoA). To mine deeper data of microbial diversity of the differences between the samples, significance test was conducted with some statistical analysis methods, including MetaStat, LEfSe, Anosim and MRPP.

### Statistical analysis

Data analysis for 16S rRNA amplicon sequencing has been described in preceding paragraphs. Other data are analyzed using GraphPad 7.0 software. If the data conformed to a normal distribution, one-way ANOVA was used to compare the means of multiple samples. The LSD method was used to compare the groups with homogeneous variances, and the Dunnett’s T3 method was used to compare the data between groups with unequal variances. Non-parametric test such as Kruskal-Wallis test was used for non-normal distribution data, *p*<0.05 was considered statistically significant.

## Results

### LJAW exerted Anti-CINV effects in a cisplatin-induced Rat Pica model

**Figure 1:**
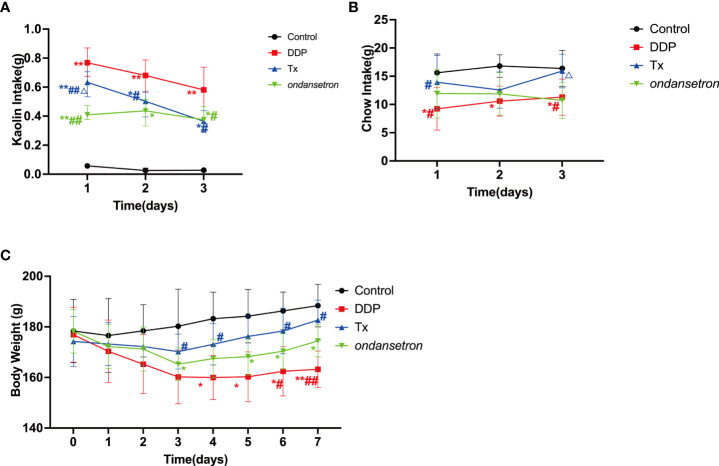
Anti-CINV Efficacy of LJAW in a Cisplatin-induced Rat-Pica Model. **(A)** Kaolin intake of rats during the first 3 days after chemotherapy (n = 10 biologically independent animals per group, data are presented as mean ± sem, normality was tested by Shapiro-Wilk normality test, p values were determined by using one-way ANOVA). **(B)** Food intake of rats during the first 3 days after chemotherapy (n = 10 biologically independent animals per group, data are presented as mean ± sem, normality was tested by Shapiro-Wilk normality test, p values were determined by using one-way ANOVA). **(C)** Dynamic changes in body weight of rats during the whole experiment (n = 10 biologically independent animals per group, data are presented as mean ± sem, normality was tested by Shapiro-Wilk normality test, p values were determined by using one-way ANOVA). (*, p < 0.05 compared with control; **, *p* < 0.01 compared with control. #, *p* < 0.05 compared with cisplatin (DDP); △, *p* < 0.05 compared with ondansetron (DDP)).

All rats showed pica behavior after cisplatin injection. We measured kaolin intake, food intake and body weight every 24 hours. Cisplatin induced substantial increased kaolin intake ranging from 0.53g to 1.09g during the first 24 hours after chemotherapy compared with vehicle control(saline) injected rats (cisplatin vs. vehicle-control, 0.78 ± 0.032g vs. 0.056 ± 0.001g, *p*<0.01). LJAW and ondansetron significantly reduced cisplatin-induced kaolin intake on the first day after cisplatin injection compared with cisplatin treated rats(cisplatin, LJAW and ondansetron, 0.78 ± 0.032g, 0.61 ± 0.22g and 0.42 ± 0.034g, respectively; LJAW vs. cisplatin, *p*<0.01; LJAW vs. ondansetron, *p*<0.05, ondansetron vs. cisplatin, *p*<0.01). Kaolin consumption was significantly decreased on the second day in LJAW and ondansetron treated mice compared with cisplatin-only treated rats, but no significant difference was observed in LJAW and ondansetron treated rats (cisplatin, LJAW and ondansetron, 0.65 ± 0.16g, 0.49 ± 0.12g and 0.44 ± 0.13g, respectively; LJAW vs. cisplatin, *p*<0.05; LJAW vs. ondansetron, *p*>0.05, ondansetron vs. cisplatin, *p*<0.05). On the third day, the kaolin intake was 0.53 ± 0.27g, 0.34 ± 0.11g and 0.38 ± 0.15g in cisplatin-, LJAW- and ondansetron-treated rats (LJAW vs. cisplatin, *p*<0.05; LJAW vs. ondansetron, *p*>0.05; ondansetron vs. cisplatin, *p*<0.05) **(** **Figure 1A**). Moreover, we observed that rats in the cisplatin group consumed much less chow within 72 hours after cisplatin injection compared with the Control rats (24hours, cisplatin vs. control, 9.23 ± 3.77g vs. 15.63 ± 3.32g, *p*<0.05; 48hours, cisplatin vs. control, 9.23 ± 3.77g vs. 15.63 ± 3.32g, *p*<0.05; 72hours, cisplatin vs. control, 9.23 ± 3.77g vs. 15.63 ± 3.32g, *p*<0.05). Interestingly, LJAW significantly increased the food intake on the first and third day after chemotherapy compared with cisplatin-only treated rats(chow consumption on the first day(cisplatin, LJAW and ondansetron), 9.23 ± 3.77g, 13.94 ± 4.37g, 11.94 ± 4.3g, LJAW vs. cisplatin, *p*<0.05; LJAW vs. ondansetron, *p*>0.05;ondansetron vs. cisplatin, *p*>0.05; chow consumption on the second day(cisplatin, LJAW and ondansetron), 10.60 ± 2.67g, 12.56 ± 3.21g, 11.88 ± 3.80g, LJAW vs. cisplatin, *p*>0.05; LJAW vs. ondansetron, *p*>0.05; ondansetron vs. cisplatin, *p*>0.05; chow consumption on the third day(cisplatin, LJAW and ondansetron), 11.30 ± 3.20g, 15.92 ± 2.98g, 10.72 ± 3.21g, LJAW vs. cisplatin, *p*<0.05; LJAW vs. ondansetron, *p*<0.05; ondansetron vs. cisplatin, *p*>0.05). However, increased food intake was not observed in the ondansetron-treated rats compared with cisplatin only treated rats (**Figure 1B**). Similarly, body weight of cisplatin or ondansetron treated rats decreased dramatically 24 hours after injection of cisplatin. Body weight of all rats started to recover 72 hrs. after the dosage of cisplatin. However, body weight loss was not present in LJAW-treated rats compared with rats in the Control group (**Figure 1C**).

### LJAW modulated cisplatin-induced gut microbiome alteration

**Figure 2:**
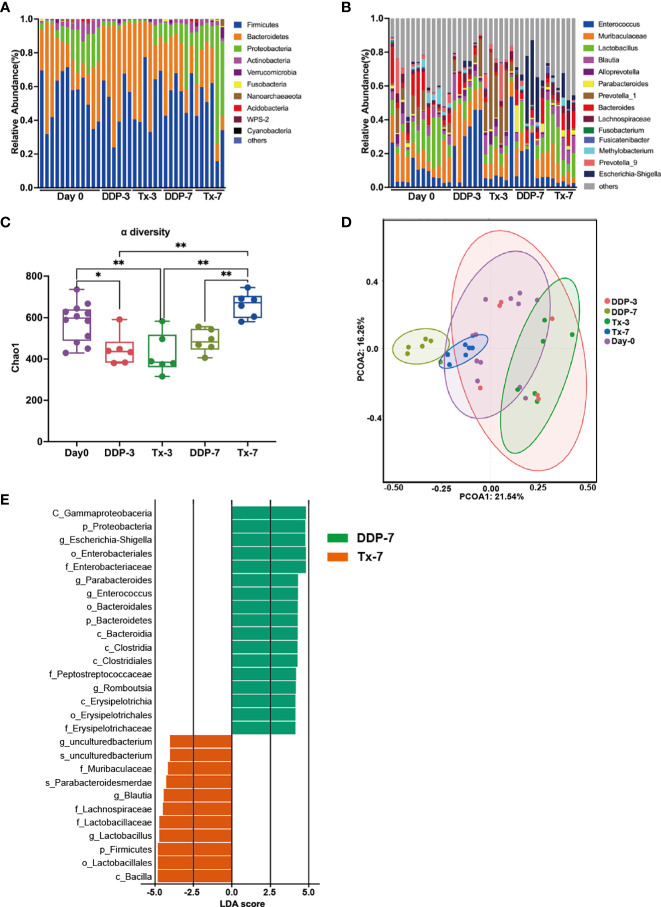
Effects of LJAW on the Gut Microbiome in Cisplatin-induced Rat-Pica Model. **(A)** Stacked bar plot of common bacterial taxa (>0.1% abundance) at the phylum level for fecal samples(Day0, n = 12; DDP-3, n = 6; Tx-3, n = 6; DDP-7, n=6; Tx-7, n=6) by 16S rRNA sequencing. **(B)** Stacked bar plot of common bacterial taxa (the top 14) at the genera level for fecal samples(Day0, n=12; DDP-3, n = 6; Tx-3, n = 6; DDP-7, n = 6; Tx-7, n = 6). **(B)** Chao richness index of fecal samples collected before, 3- and 7-days after treatment. (Day0, n = 12; DDP-3, n = 6; Tx-3, n = 6; DDP-7, n = 6; Tx-7, n = 6. The bars represent the median and the distribution of Chao1 index. *p* values were determined using Kruskal-Wallis test. * *p*<0.05, ** *p*<0.01). **(C)** Alpha diversity of fecal samples collected before, 3- and 7-days after treatment(Day0, n=12; DDP-3, n=6; Tx-3, n=6; DDP-7, n=6; Tx-7, n=6. * p< 0.05, ** p<0.01). **(D)** Principal coordinate analysis of fecal samples (n = 30) by response using Weighted UniFrac distances. **(E)** LDA scores calculated for differentially abundant taxa in the fecal microbiomes of different groups and different time points. Length indicates the effect size associated with a taxon. (LDA score > 4).

Microbiome profiling was performed to explore composition of intestinal microbiome in cisplatin- and LJAW- treated rats before (Day 0), 3 days and 7 days after cisplatin injection. First, we compared the gut microbiota diversity among groups using the Chao index. A significant decline in alpha diversity was observed in the feces collected 3 or 7 days after cisplatin injection compared with the feces collected 3 or 7 days after LJAW treatment (**Figure 2C**). Then, principal coordinates analysis was performed to explore similarities between these two datasets. A notable clustering effect by cisplatin injection was exerted in the intestinal microbiome of rats (**Figure 2D**). However, no clustering effect was observed in samples collected after 7 days’ treatment of LJAW compared with samples collected prior treatment. Next, we sought to further examine the composition changes of microbiome induced by cisplatin as well as LJAW. Results showed that all rats were dominated by *Bacteroidetes* and *Firmicutes* at the phylum level. However, the abundance of *Bacteroidetes* at the phylum level was increased 3 days after cisplatin injection compared to the abundances in the samples collected before injection (**Figure 2A**). At the genus level, the bacterial composition in feces collected before cisplatin injection was dominated by *Enterococcu*s, *Muribaculaceae*, and *Lactobacillus* but devoid of Escherichia-Shigella (**Figure 2B**). Cisplatin injection caused an elevated abundance of *Enterococcu*s *and Escherichia-Shigella* on both the 3^rd^ and 7^th^ day after cisplatin injection, but decreased abundance of *Lactobacillus* (**Figures 2B**). By contrast, the intestine microbiome was dominated by ASVs belong to *Muribaculaceae, Lactobacillus, Blautia and Bacteroides, and fewer ASVs belonging to the Enterococcu*s and *Escherichia-Shigella* after 3- or 7-days’ treatment of LJAW (**Figures 2B**). To further investigate these findings, we conducted high-dimensional class comparisons *via* linear discriminant analysis of effect size (LEfSe), which again validated differentially abundant bacteria in the fecal microbiome of cisplatin- and LJAW-treated rats, with *Escherichia-Shigella, Parabacteroides and Enterococcu*s genera enriched in DDP-7 and *Blautia* and *Lactobacillus* enriched in Tx-7 (**Figure 2E**).

*Escherichia-Shigella* was well documented as harmful bacteria which involved in the bowel inflammation and carcinogenesis of intestine (Lang et al., 2020; Fan et al., 2021). *Lactobacillus* was identified as probiotics and was prescribed to treat inflammatory bowel diseases *(* Wu et al., 2020 *).* Consistently, *Blautia* could also exert an anti-inflammatory function in the intestine and has been named a new functional genus with potential probiotic properties (Davrandi et al., 2021; Liu et al., 2021). Moreover, an experimental survey mimicking autism syndrome reported a 50% reduction in 5-HT in both small and large intestine mucosal 5-HT levels with a certain correlation to the abundance of *Blautia*, indicating that *Blautia* might participate in the synthesis or release of 5-HT (Agus et al., 2018). Here, we evaluated both the 5-HT level and the expression of TPH1, a rate-limiting enzyme of 5-HT, and the levels of proinflammatory cytokines and the expression of possible molecules involved in microbe-mediated inflammation.

### LJAW inhibited the synthesis of 5-HT in Rat Jejunum tissues

It has been reported that a variety of neurotransmitters and their receptors are involved in the development of CINV, and serotonin (also named 5-hydroxytryptamine) plays a key role in the pathogenic process of CINV (Marx et al., 2017). Cytotoxic medicine such as cisplatin could cause the release of 5-HT in enterochromaffin cells in the intestinal mucosal barrier, the elevated 5-HT that is released stimulates the 5-HT receptors in both the central and peripheral nervous systems and regulates the emetic pathways (Zhong et al., 2017).

**Figure 3:**
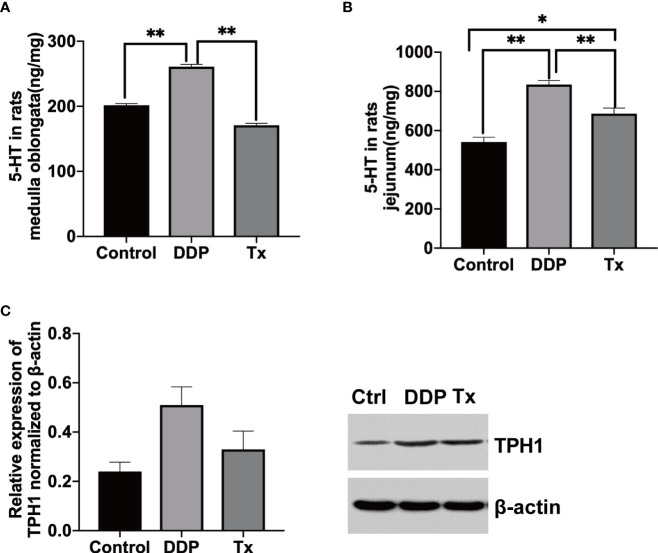
LJAW Downregulated the Secretion of 5-HT in Both the Jejunum and Medulla Oblongata by Inhibiting THP1 in Rats. **(A)** 5-HT concentration in rat jejunum tissue(n = 5, data are presented as mean ± sem, normality was tested by Shapiro-Wilk normality test, p values were determined by using one-way ANOVA, *, *p* < 0.05, **, *p* < 0.01). **(B)** 5-HT concentration in rat medulla oblongata tissue(n=5, data are presented as mean ± sem, normality was tested by Shapiro-Wilk normality test, *p* values were determined by using one-way ANOVA, *, *p* < 0.05, **, *p* < 0.01). **(C)** Expression of TPH1 in rat jejunum tissue(n=3, data are presented as median with interquartile range, normality was tested by Shapiro-Wilk normality test, *p* values were determined by using one-way ANOVA, *, *p* < 0.05, **, *p* < 0.01).

To elucidate the possible mechanisms involved in LJAW-elicited anti-CINV efficacy, we measured the concentration of 5-HT in both rat jejunum and medulla oblongata tissues. As shown in **Figure 3A**, the level of 5-HT significantly increased in both jejunum and medulla oblongata tissues 3 days post-cisplatin injection compared with vehicle-control (5-HT levels in rats jejunum (Control, DDP and TX), 541 ± 59ng/mg, 835 ± 48 ng/mg and 687 ± 67 ng/mg, Control vs. DDP, *p*<0.01; Control vs. Tx, *p*>0.05; DDP vs. Tx, *p*<0.01. 5-HT in rats medulla oblongata (Control, DDP and TX), 270 ± 9ng/mg, 260 ± 9ng/mg and 170 ± 6ng/mg, Control vs. DDP, *p*<0.01; Control vs. Tx, p<0.05; DDP vs. Tx, *p*<0.01). The 5-HT levels were also significantly higher in the LJAW-treated rats than in the vehicle-control treated rats. However, it was still much lower compared with that of DDP-3 in rat jejunum tissue (**Figures 3A, B**). Then, we measured the expression of TPH1, a key enzyme participating in the synthesis of 5-HT in jejunum tissues, using western blotting. TPH1 expression was slightly upregulated 3 days after cisplatin injection. Conversely, it was downregulated in LJAW-treated rats (**Figure 3C**).

### LJAW protected the intestinal mucosal barrier in cisplatin-induced pica in rats

As most 5-HT-producing enterochromaffin cells reside in the jejunum, necrosis of enterochromaffin induced by cisplatin could also promote the release of 5-HT (Linan-Rico et al., 2016). We evaluated the integrity of the jejunum mucosa with HE staining and measured the expression of the tight junction-associated proteins occludin and ZO-1 in LJAW-treated rat jejunum tissue by IHC.

**Figure 4:**
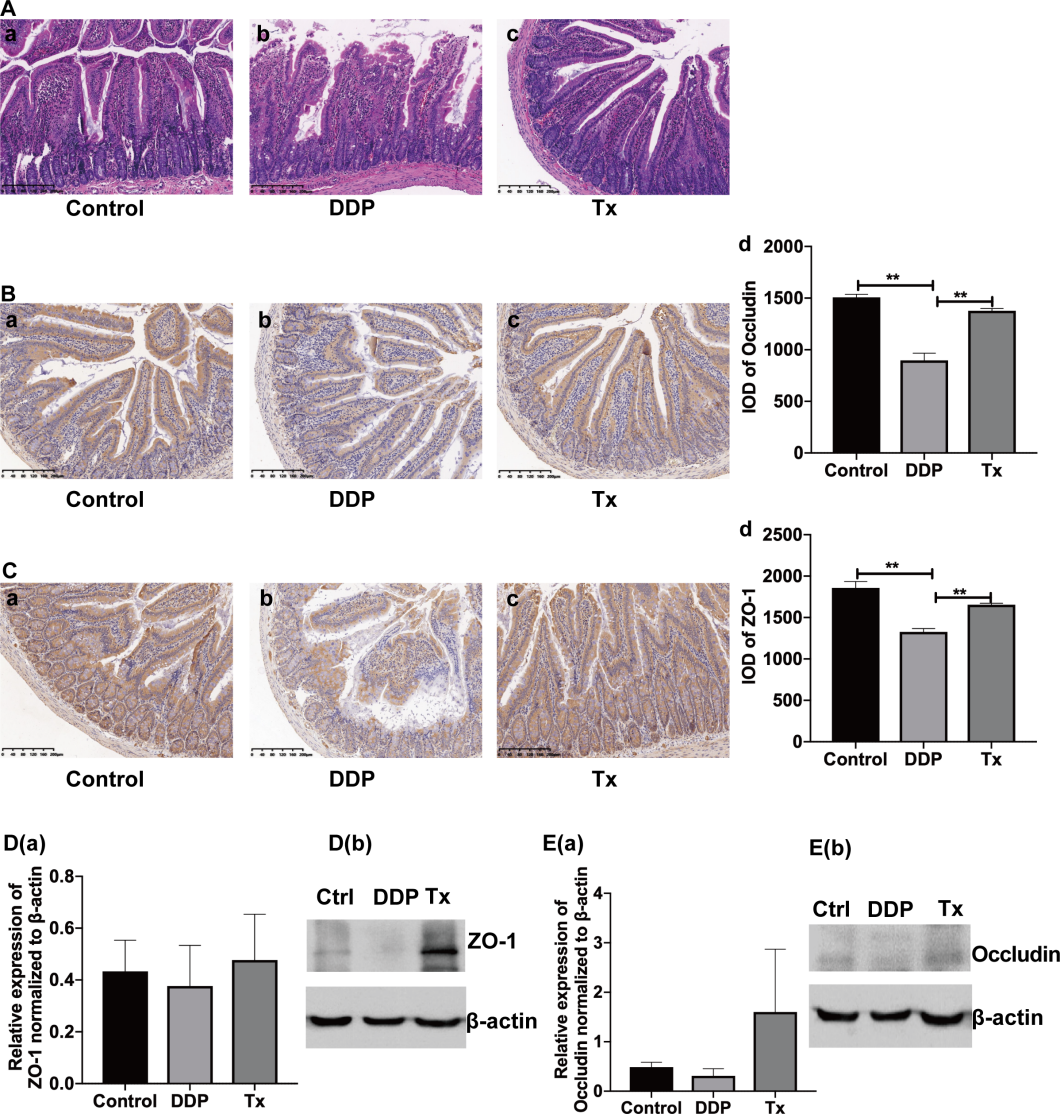
Protective Effect of LJAW on the Intestinal Mucosal Barrier in Cisplatin-induced Pica in Rats. **(A)** HE staining of representative intestine tissue sections obtained from rats treated with **(A)** vehicle control, **(B)** cisplatin or **(C)** cisplatin and LJAW. **(B)** Immunohistochemistry staining of occludin in jejunum tissue **(A–C)**. The expression of occludin in jejunum tissue was quantified by integrated optical density (IOD) **(D)**(n = 3, data are presented as mean ± sem, normality was tested by Shapiro-Wilk normality test, p values were determined by using one-way ANOVA, **, *p* < 0.01). **(C)** Immunohistochemistry staining of ZO-1 in jejunum tissue **(A–C)**. The expression of ZO-1 in jejunum tissue quantified by integrated optical density (IOD) **(D)** (n=3, data are presented as mean ± sem, normality was tested by Shapiro-Wilk normality test, p values were determined by using one-way ANOVA, **, *p* < 0.01). **(D)** Protein expression of ZO-1 in jejunum tissue. **D(a)** western blots showing total levels of ZO-1 in Control-, DDP-, and LJAW-treated rats jejunum, **D(b)** Bar graphs showing mean ZO-1 levels determined by densitometey(n=3, data are presented as mean ± sem, normality was tested by Shapiro-Wilk normality test, p values were determined by using one-way ANOVA). **(E)** Protein expression of Occludin in jejunum tissue. **E(a)** western blots showing total levels of Occludin in Control-, DDP-, and LJAW-treated rats jejunum, **E(b)** Bar graphs showing mean Occludin levels determined by densitometry (n=3, data are presented as mean ± sem, normality was tested by Shapiro-Wilk normality test, *p* values were determined by using one-way ANOVA).

The HE staining showed that there was no disruption of the jejunal mucosa before cisplatin injection. Additionally, the morphology of the jejunal glands and villi was complete. Furthermore, the epithelial cells were neatly arranged, and cell degeneration and cell necrosis were not observed, as shown in **Figure 4A(a)**. Disrupted mucosa and shortened, sparse, or even lost villi were observed in the jejunum tissues of rats treated 7 days after cisplatin injection **(Figure 4A(b)**. As shown in **Figure 4A(c)**, the villi were normal in shape, and attenuated mucosa disruption was observed in LJAW treated rat’s intestine tissues.

Then, we sought to determine whether the intestinal mucosa barrier protective effect of LJAW was mediated by tight junction-associated proteins. We measured the expression of both occludin and ZO-1 in rat jejunum tissue through IHC staining and western blotting. Elevated occludin and ZO-1 protein expression was observed in the LJAW-treated rats compared with the cisplatin-treated rats (**Figures 4B–E**).

### LJAW inhibited the expression of proinflammatory cytokines and related proteins

**Figure 5:**
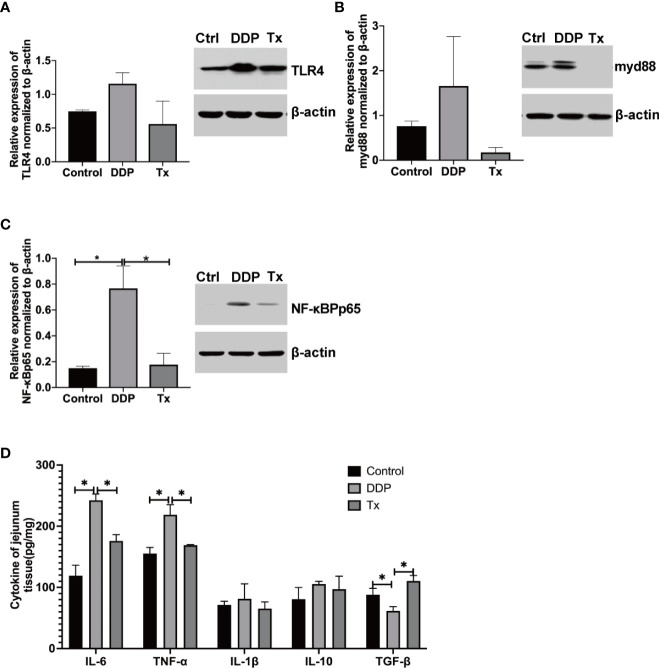
LJAW Downregulated Proinflammatory Cytokine Expression in Jejunum Tissues. **(A**–**C)** western blotting of the expression of the proinflammatory cytokine-related proteins TLR4, myd88, and NF-κB p56 in jejunum tissues of LJAW-treated rats, cisplatin-treated rats and vehicle control-treated rats(n = 3 in each group, A(a), B(a), C(a),western blots showing total levels of TLR4, myd88, and NF-κB p56 in Control-, DDP-, and LJAW-treated rats jejunum; A(b), B(b), C(b), Bar graphs showing mean TLR4, myd88, and NF-κB p56 levels determined by densitometry(n = 3, data are presented as mean ± sem, normality was tested by Shapiro-Wilk normality test, *p* values were determined by using one-way ANOVA, * *p* < 0.05). **(D)** Cytokine expressions of IL-6, TNF-α, IL-1β, IL-10, and TGF-β in LJAW-treated rats, cisplatin or vehicle control-treated rats jejunum tissues. (n = 6, Data are presented as the mean ± sem. normality was tested by Shapiro-Wilk normality test, *p* values were determined by using one-way ANOVA *p < 0.05).

Bowel inflammation has already been validated in cisplatin-treated rat intestine tissues (Perales-Puchalt et al., 2018). In our study, we found that *Escherichia-Shigella, and Enterococcu*s genera was enriched in rat feces collected 7 days after cisplatin injection, while *Blautia* and *Lactobacillus* genera was enriched in fecal samples collected 7 days after LJAW dosage, as shown in **Figure 2E**. Thus, ongoing inflammatory processes might be partially blocked or alleviated in LJAW-treated rat intestines. To further verify our hypothesis, we measured the pro-inflammatory cytokines and mucosa repair-related cytokines or chemokines in the rat jejunum with ELISA kits. The results showed that IL-6 and TNF-α were significantly elevated 72 hours after cisplatin injection; however, they were dramatically downregulated in LJAW-treated rats (**Figure 5D**). Then, to confirm whether the downregulated inflammation level in LJAW-treated rats was mediated by intestinal microbes through signaling to NF-κB by Toll-like receptors (TLRs), we performed western blotting to measure the protein expressions of TLR4, NF-κB and myd88 in rat jejunum tissues. As speculated, cisplatin induced upregulated protein expression of TLR4, myd88 and NF-κB compared with the control rats (**Figures 5A–C**). LJAW downregulated the expression of TLR4, myd88 and NF-κB in jejunum tissues compared with DDP treated rats.

## Discussion

Few side effects of cancer treatment are more feared by patients than CINV, especially in patients undergoing highly emetogenic regimens or multiagent chemotherapy. The combined application of medicines with different mechanisms in anti-CINV is recommended by different guidelines to manage CINV (Razvi et al., 2019). However, patients are still suffering from nausea, which would also induce loss of appetite (Ng et al., 2015). There are unmet needs in patients treated with chemotherapy. LJAW has been proven to be effective in clinical studies. Here, we validated the anti-CINV efficacy of LJAW in a cisplatin-induced rat pica model. As presented in **Figure 1A**, LJAW decreased the kaolin intake induced by cisplatin injection of rats, which is an alternative way to evaluate the severity of nausea and vomiting in rats, revealing that LJAW attenuated CINV in rat-pica model. In contrast to conventional anti-CINV agents, LJAW also improved the appetite of rats with increased chow consumption compared with cisplatin-only treated rats (**Figure 1B**). Therefore, no body weight loss was observed in the rats treated with LJAW. The above results indicate that LJAW not only alleviated CINV but also improved the appetite of rats and guaranteed stable body weight gain during chemotherapy.

As gastrointestinal disorders, for instance, diarrhea or constipation frequently occur in patients receiving chemotherapy, and investigations have shown that the gut microbiome plays a pivotal role in mediating chemotherapy-induced side effects, including nausea and vomiting (Hong et al., 2019; Crowder et al., 2021). Cisplatin is known to inhibit the growth of both Gram-negative and Gram-positive bacterial strains, such as some Bacillus and E. coli, and may induce dysbiosis (Joyce et al., 2010). The gut microbiota is involved in the modulation of other common side effects of cisplatin, such as cytotoxicity and mucositis. In this study, we observed that cisplatin injection indeed resulted in an alteration of the gut microbiome in rats with lowered alpha diversity, elevated abundance of *Bacteroidetes* at the phylum level and further enriched *Escherichia-Shigella, and Enterococcu*s at the genus level (**Figures 2A, B**). Numerous studies have shown the efficacy of TCM in treating CINV (Han et al., 2016; Thamlikitkul and Soparattanapaisarn, 2016), and the application of ginger has been recommended as an alternative way to manage CINV according to certain guidelines (Razvi et al., 2019). Mechanisms including decreasing bowel movement and downregulating 5-HT levels in the intestine and medulla oblongata have been shown to participate in the anti-CINV efficacy of TCM (Marx et al., 2017; Yi et al., 2019). However, few studies have provided insight into the role of the intestinal microbiome in mediating the anti-CINV effect of TCM. Here, we measured the gut microbiome before and after LJAW intervention by 16S rRNA sequencing. As described in the previous paragraphs, LJAW treatment resulted in an elevated alpha diversity compared with cisplatin-only treated rats. Further taxonomic analysis revealed that cisplatin induced enrichment of *Escherichia-Shigella and Enterococcu*s at the genus level while samples collected in LJAW treated rats were enriched in *Blautia* and *Lactobacillus* uncovered by taxonomic cladogram from LEfSe analysis (**Figure 2E**). These results indicated that LJAW played an important role in maintaining intestine microbiome balance.

*Enterococcu*s is a representative genera that belongs to the family of *Enterococcaceae.* It was reported to exerts a proinflammatory role in IBD patients (Kowalska-Duplaga et al., 2020). An animal study showed that the concentrations of proinflammatory cytokines, such as IL-6, TNF-α, IL-17a, and IL1β, were positively correlated with the abundance of *Enterococcaceae* (Seishima et al., 2019). *Escherichia-Shigella* belongs to the family of *Enterobacteriaceae*. It’s been documented to be the most important enteric pathogens causing bacillary dysentery (Devanga Ragupathi et al., 2017). Thus, it might play a role in cisplatin induced stool consistency change. Mechanism study has proved that *Escherichia-Shigella* was able to produce Lipopolysaccharide(LPS) that triggering the activation of intestine inflammation *via* binding with the complex CD14, myeloid differentiation protein 2, and Toll-like receptor 4(TLR4) (Paciello et al., 2013). By the contrast, the genus *Blautia* notably includes anaerobic intestinal commensal organisms within the bacterial class *Clostridia* belonging to the phylum *Firmicutes* (Liu et al., 2008; Park et al., 2013) and was demonstrated to exert anti-inflammatory effects on a variety of diseases (Benítez-Páez et al., 2020). For instance, *Blautia* was reported to be associated with a localized anti-inflammatory effect, primarily in the intestinal tract, without systemic immune suppression in graft-versus-host disease (Jenq et al., 2015). When stressed mice were treated with minocycline, the observed gut microbiota changes included an increase in the relative abundance of *Akkermansia* spp. and *Blautia* spp., which are compatible with the beneficial effects of attenuated inflammation and rebalance of gut microbiota (Wong et al., 2016). Moreover, *Lactobacillus* has been reported to inhibit inflammation *via* TLR4/MYD88/NF-κB axis (Li et al., 2020). The microbiota such as *Escherichia-Shigella* could interact with the host immune system *via* pattern recognition receptors such as toll-like receptors (TLRs) on epithelial cells by microbe-derived pathogen-associated molecular patterns such as LPS to activate myd88-dependent signaling, ultimately inducing the production of proinflammatory cytokines (Dutta and Lim, 2020). Our findings are consistent with what has been reported in the literature. Inflammation-related cytokines, especially IL-6 and TNF-α, were significantly increased after chemotherapy (**Figure 4D**) and were downregulated by LJAW treatment.

Apart from inflammation, the secretion of serotonin induced by chemotherapy played a pivotal role in CINV. Cisplatin can cause loss of integrity in the intestinal mucosa by binding to DNA, thus impairing the DNA replication of rapidly proliferating epithelial cells. This damage results in a breach of mucosal barriers and subsequently allows microbes to come in close proximity to epithelial cells, thus triggering local and systemic immune responses (Taur and Pamer, 2016). Moreover, enterochromaffin cell damage occurs under these conditions and promotes the release of 5-HT, which interacts with the peripheral and central nervous systems to cause nausea and vomiting. In other words, inflammation might improve the 5-HT level and cause more severe clinical symptoms. The intestinal microbiome also participated in the synthesis of 5-HT. Regardless of the anti-inflammatory properties, a decrease in *Blautia* abundance has been reported in the mucosal adherent microbiota of colorectal cancer patients. Interestingly, *Blautia* was also reported to be involved in tryptophan metabolism and indirectly affects its synthesis in psychological diseases such as autism and depression (Golubeva et al., 2017; Agus et al., 2018; Liu et al., 2020). We observed that the mucosa breached and that tight junction-associated proteins, including ZO-1 and occludin, were downregulated after chemotherapy (**Figure 4**). As predicted, LJAW showed a mucosa barrier protective effect (**Figure 4A**) and upregulated the expression of both ZO-1 and occludin compared with cisplatin-only treated rats (**Figures 4B–D**). Meanwhile, the 5-HT levels were elevated in both the jejunum and medulla oblongata tissues of cisplatin-treated rats compared with tissues collected before chemotherapy (**Figures 3A, B**). Moreover, we found that the rate-limiting enzyme TPH1 was upregulated after cisplatin injection but downregulated in LJAW-treated rats. These results indicate that LJAW not only inhibited the secretion by protecting the mucosal barrier but also inhibited the synthesis of serotonin, possibly *via* gut microbiome-mediated THP1 expression.

In conclusion, LJAW exerted anti-CINV efficacy in a cisplatin-induced rat pica model, possibly through gut microbiome-mediated anti-inflammation and anti- serotonin synthesis functions. However, we did not conduct the experiment in germ-free rats to further validate the exact role of enriched genera including *Escherichia-Shigella*, *Enterococcu*s*, Blautia* and *Lactobacillus* in LJAW-elicited anti-CINV effects. Further studies are needed to investigate this hypothesis in depth using germ-free rats to draw definitive conclusions about the potential and differential roles of the microbiome in the management of CINV by LJAW. Next, we also sought to determine how LJAW could modulate gut microbiome and the exact compounds that participated in gut microbiome modulation.

## Data availability statement

The datasets presented in this study can be found in online repositories. The name of the repository and accession number can be found below: NCBI; PRJNA816481.

## Ethics statement

The animal study was reviewed and approved by Implementing Principles of Laboratory Animal Ethics of Xiyuan Hospital, China Academy of Chinese Medical Sciences.

## Author contributions

DC: manuscript drafting, study design, and statistical analysis. YG: performed experiments. DC and YG contributed equally to this study. YY: study design and administration support. All authors contributed to the article and approved the submitted version.

## Funding

This study is supported by Foundation for Young Scientist of China-Japan Friendship Hospital (2019-2-QN-63), Natural Science Foundation of Beijing Municipality (7214295), personnel training program of China-Japan Friendship Hospital elite project (ZRJY2021-TD05), National Natural Science Foundation of China(81904138 and 82104599) and National key research and development program(2017YFC1700604).

## Acknowledgments

We thank Dr Peiying Yang from The University of Texas MD Anderson Cancer Center in the US for her guidance of the study design, as well as Dr Cao Zou and Xia Wang who helped with experiment conduction.

## Conflict of interest

The authors declare that the research was conducted in the absence of any commercial or financial relationships that could be construed as a potential conflict of interest.

## Publisher’s note

All claims expressed in this article are solely those of the authors and do not necessarily represent those of their affiliated organizations, or those of the publisher, the editors and the reviewers. Any product that may be evaluated in this article, or claim that may be made by its manufacturer, is not guaranteed or endorsed by the publisher.
